# A Cross-Sectional Study of Short-Video Viewing and Young Adult Health: Dose–Response Relationships with Sleep, Dream Anxiety, Ocular Surface, and Quality of Life

**DOI:** 10.3390/healthcare14172748

**Published:** 2026-08-28

**Authors:** Xiaoyu Wang, Yanmei Zeng, Xiangyi Liu, Wenjuan Yang, Yi Liu, Xu Chen, Yixin Wang, Yan Lou, Yi Shao

**Affiliations:** 1 Department of Ophthalmology, The First Affiliated Hospital of Nanchang University, Nanchang University, Nanchang 330000, China; 2 Department of Ophthalmology and Visual Sciences, Maastricht University, 6200 MA Maastricht, The Netherlands; 3 Singapore National Eye Centre, Singapore 168751, Singapore; 4 School of Medical Information and Engineering, Southwest Medical University, Luzhou 646000, China; 5 Department of ophthalmology, Shanghai General Hospital, Shanghai Jiao Tong University School of Medicine, Shanghai 200000, China

**Keywords:** short-video viewing, dry eye, dream anxiety, sleep duration, dose–response, mobile phone addiction

## Abstract

**Background:** Short-video platforms (e.g., Douyin, TikTok) have gained immense popularity among young adults, yet their addictive potential and health consequences remain understudied. Unlike gaming addiction, which has been linked to myopia and mental distress, short-video viewing may pose distinct risks due to its fragmented, high-frequency, and pre-sleep usage patterns. This study aimed to investigate the relationships between short-video viewing and multifaceted health outcomes in Chinese young adults, with a focus on dose–response relationships. **Methods:** A cross-sectional survey was conducted among 328 young adults aged 20–33 years. Participants were categorized into a non-addicted group (daily short-video viewing < 0.5 h, *n* = 164) and a short-video viewing group (daily viewing ≥ 1 h, *n* = 164), further divided by duration: 1–2 h (*n* = 96), 3–4 h (*n* = 29), 5–6 h (*n* = 22), and >6 h (*n* = 17). Outcome measures included the Hospital Anxiety and Depression Scale (HADS), Van Dream Anxiety Scale (VDAS), 36-Item Short-Form Health Survey (SF-36), Ocular Surface Disease Index (OSDI), Internet Addiction Test (IAT), Chinese Internet Addiction Scale—Revised (CIAS-R), and Mobile Phone Addiction Index (MAPI). Linear regression, one-way ANOVA with Tukey post hoc comparisons (or Kruskal–Wallis tests with Dunn’s test for non-normal variables), and dose–response curve fitting were applied. Segmented regression was used to explore potential dose–response patterns. **Results:** Compared with the non-addicted group, the short-video viewing group had significantly shorter sleep duration, higher HADS, VDAS, OSDI, IAT, CIAS-R, and MAPI scores, and lower SF-36 scores (all *p* < 0.0001). Clear dose–response relationships were observed: as daily viewing duration increased, sleep duration decreased linearly (r = −0.73, *p* < 0.001), while OSDI (r = 0.89, *p* < 0.001) and VDAS (r = 0.68, *p* < 0.001) increased. Among participants viewing >6 h/day, all (100%) had moderate-to-severe dry eye (OSDI ≥ 23), and 71% (12/17) had probable anxiety/depression (HADS ≥ 11). Notably, MAPI showed the strongest correlation with VDAS (r = 0.77, *p* < 0.001), indicating that mobile phone addiction was closely associated with nightmare-related anxiety. The SF-36 physical component summary (PCS) was negatively correlated with daily short-video viewing duration (r = −0.62, *p* < 0.001). Exploratory threshold analyses suggested that viewing > 3 h/day was associated with greater severity of sleep and ocular surface complaints. The dose–response thresholds identified in this study are exploratory, as the number of participants in the higher-duration categories was limited (3–4 h: *n* = 29; 5–6 h: *n* = 22; >6 h: *n* = 17). These breakpoints should be interpreted with caution and validated in future large-scale studies. **Conclusions:** Short-video viewing is associated in a dose-dependent manner with sleep quality, ocular surface health, and psychological well-being, with effect sizes generally comparable to, and in some domains (e.g., dry eye and dream anxiety) somewhat larger than, those reported for gaming addiction. The strong association between mobile phone addiction (MAPI) and dream anxiety (VDAS) highlights the distinctive association pattern of pre-sleep short-video use. Future interventional studies are warranted to determine the optimal duration limit. Our exploratory thresholds (approximately 3.2 h/day for sleep and 4.0 h/day for OSDI) provide preliminary reference points, but the specific target duration remains to be established through adequately powered studies designed to compare multiple cut-off levels.

## 1. Introduction

The digital age has witnessed a dramatic shift in young adult leisure activities from traditional video games to short-video platforms such as Douyin (TikTok) and Kuaishou. These platforms use algorithm-driven, infinite-scroll interfaces that provide intermittent, unpredictable rewards, which are highly effective in capturing attention and promoting compulsive use [1,2]. This continuous consumption loop shapes more than just usage habits; the rapid delivery of emotionally charged content on platforms like TikTok actively drives profound discourse shifts and ideological signalling, altering how users process public information and conceptualise risk during leisure time [3]. Unlike gaming, which typically involves goal-oriented, task-completion cycles, short-video consumption is characterised by fragmented viewing, frequent task-switching, and high emotional arousal—features that may uniquely affect sleep, ocular surface, and mental health [4].

We distinguish short-video viewing from general screen time, which includes passive TV viewing, computer work, and gaming, and from general social media use, which is primarily interaction-based. Short-video platforms are characterised by three features: (1) algorithm-driven infinite scrolling that delivers unpredictable, emotionally charged content; (2) rapid scene changes every 3–15 s that suppress blinking and induce sustained attentional capture; and (3) a predominantly passive consumption mode with minimal user-generated content or social interaction. These features may confer risks distinct from those associated with other screen activities.

Global prevalence estimates show that over 60% of Chinese young adults use short-video apps daily, with 15–20% exhibiting signs of addictive use [5]. The World Health Organization has not yet classified short-video viewing as a distinct disorder, but its phenomenological overlap with internet gaming disorder (IGD) and compulsive sexual behaviour disorder has prompted calls for research [6]. However, most existing studies have focused on social media or gaming, leaving a gap regarding the specific health impacts of short-video viewing.

Our previous work [7] demonstrated that gaming addiction is associated with myopia, dry eye, anxiety, depression, and reduced quality of life, with notable gender differences, with males more affected. Short-video viewing may differ in three key aspects: (1) viewing distance and blinking—handheld devices at closer range and faster scene cuts may suppress blink rate more severely, accelerating tear film instability [8]; (2) pre-sleep usage—short-videos are often consumed in bed before sleep, exposing young adults to blue light and cognitively arousing content that delays sleep onset and disrupts REM sleep [9]; (3) psychological mechanism—the passive, algorithm-guided consumption may foster a sense of time loss and helplessness, potentially increasing dream-related anxiety [10]. While short-video viewing and gaming share common pathways such as screen time, blue light exposure, and reduced blinking, we argue that short-video platforms introduce several unique risk factors: (1) the average viewing distance is closer due to handheld device use; (2) the rapid scene cuts and unpredictable content suppress blink rates more severely than goal-oriented gaming; and (3) the pre-sleep usage pattern is more prevalent for short-videos than for gaming. These distinctions, rather than a categorical difference, form the basis of our hypothesis.

Therefore, we hypothesized that short-video viewing would be associated with: (i) shorter sleep duration in a dose-dependent manner; (ii) higher ocular surface disease index (OSDI) scores, possibly exceeding those seen in gaming addiction; (iii) elevated dream anxiety (VDAS), especially linked to mobile phone addiction (MAPI); and (iv) reduced physical and mental quality of life (SF-36). We expected nonlinear dose–response relationships based on the ‘Goldilocks hypothesis’ [11], which suggests that moderate screen time may be benign while excessive use becomes harmful. Furthermore, studies on behavioural addictions have observed threshold effects, where health outcomes deteriorate disproportionately beyond certain usage levels. We therefore employed segmented regression to explore potential breakpoints, though these analyses were pre-specified as exploratory. The primary objective of this study was to examine the association between daily short-video viewing duration and multiple health outcomes. A secondary objective was to explore potential dose–response thresholds using segmented regression. To test these hypotheses, we conducted a cross-sectional study using multiple validated scales, including the rarely used Chinese Internet Addiction Scale (CIAS-R) and Mobile Phone Addiction Index (MAPI), to capture the broader construct of smartphone dependence. We acknowledge that the literature on screen time and health outcomes is not unanimous; some studies have reported attenuated or null relationships after controlling for confounders such as physical activity and socioeconomic status. However, these studies typically aggregate all screen types and may obscure the specific effects of short-video viewing, which is characterised by passive consumption and pre-sleep use—patterns that may confer distinct risks.

## 2. Materials and Methods

### 2.1. Participants and Procedure

Participants were recruited via convenience sampling through social media platforms (WeChat and QQ) between July 2024 and January 2025. An online questionnaire link was distributed through academic and professional networks, as well as community group chats. The survey was open to adults aged 20–33 years, with no restrictions on geographic region within China. To minimise selection bias, the recruitment advertisement did not mention specific hypotheses or health outcomes, instead describing the study as a general survey on lifestyle and media use. The inclusion criteria were as follows: (1) age 20–33 years; and (2) no major physical or psychiatric disorders. The exclusion criteria were as follows: incomplete responses or inconsistent answers (e.g., reported daily short-video viewing duration > 16 h). A total of 412 responses were received; after excluding 82 incomplete or invalid records, 328 participants were included. To assess potential selection bias, we compared the excluded participants (*n* = 82) with the included participants (*n* = 328) on age, gender, and region using independent *t*-tests and chi-square tests. No statistically significant differences were found (age: t = 0.67, *p* = 0.50; gender: χ^2^ = 0.21, *p* = 0.65; region: χ^2^ = 0.31, *p* = 0.58), suggesting that exclusion of incomplete responses did not introduce significant selection bias. Participants were divided into two groups (The demographic and clinical characteristics of the two groups are summarised in Table 1):

Non-addicted group (*n* = 164): self-reported daily short-video viewing < 0.5 h and IAT score < 50 (to exclude individuals with problematic internet use patterns regardless of short-video time). Short-video group (*n* = 164): daily viewing ≥ 1 h (no IAT threshold applied, thus encompassing a spectrum of addiction severity). This grouping strategy may introduce some overlap in non-addiction characteristics but was chosen to reflect real-world usage patterns while maintaining a clear non-addicted reference. The short-video group was therefore defined solely by daily short-video viewing duration, not by IAT score, and included individuals with varying levels of addiction severity. We acknowledge that the grouping strategy was asymmetric: the non-addicted group required both low self-reported viewing time (<0.5 h/day) and an IAT score < 50 to exclude any problematic internet use, whereas the short-video group was defined solely by daily viewing duration (≥1 h). This design was chosen to reflect real-world usage patterns, where individuals with high viewing time may exhibit a spectrum of addiction severity. Restricting the short-video group to only those with high IAT scores would have excluded many heavy users who do not meet the IAT threshold but still experience health consequences, thereby artificially narrowing the exposure range and limiting our ability to examine dose–response relationships across the full spectrum of viewing durations. This pragmatic decision was made to preserve variability in exposure, at the cost of some interpretative clarity regarding addiction specificity. This approach aligns with previous epidemiological studies on screen time behaviours.

We fully acknowledge that the asymmetric grouping strategy does not allow us to unequivocally attribute the observed differences specifically to short-video exposure rather than to broader internet/mobile phone addiction. The non-addicted group was defined by both low duration and low IAT, whereas the short-video group was defined by high duration regardless of IAT. Consequently, the two groups differ not only in short-video viewing time but also in overall internet addiction severity (as reflected in IAT, CIAS-R, and MAPI scores; Table 2). The sensitivity analysis stratifying the short-video group by IAT was underpowered (*n* = 7 for the low-IAT subgroup) and therefore could not fully resolve this confounding. Thus, our findings should be interpreted as associations with a composite exposure pattern (high short-video use accompanied by elevated smartphone/internet dependency) rather than as isolated effects of short-video duration per se.

No formal a priori power analysis was conducted, as the study was exploratory in nature and the effect sizes for short-video viewing on the specific outcomes were unknown at the time of study design. However, post hoc power calculations using GPower (version 3.1, Heinrich-Heine-Universität Düsseldorf, Düsseldorf, Germany) (based on the observed effect sizes for the primary comparison of OSDI between groups, Cohen’s d = 0.89) indicated that the achieved power exceeded 0.99 for two-tailed independent *t*-tests at α = 0.05. For subgroup analyses with smaller samples, we acknowledge the reduced statistical power and interpret results accordingly.

Ethical approval was obtained from the Ethics Committee of the First Affiliated Hospital of Nanchang University (approval number: 2023029, approved on 1 March 2023). All participants provided written informed consent. This study was conducted in accordance with the Declaration of Helsinki.

### 2.2. Measures

All instruments were administered in Chinese and had established reliability and validity.

Hospital Anxiety and Depression Scale (HADS) [12]: 14 items, yielding subscales for anxiety and depression; total scores ≥ 11 indicate probable clinical caseness.

Van Dream Anxiety Scale (VDAS) [13]: a 12-item scale assessing nightmare frequency, sleep disturbance due to nightmares, and difficulty returning to sleep. Total score range 0–48; higher scores indicate worse dream-related anxiety.

36-Item Short-Form Health Survey (SF-36) [14]: measures health-related quality of life across eight domains, summarized into Physical Component Summary (PCS) and Mental Component Summary (MCS). Scores range from 0–100, with higher scores indicating better health-related quality of life.

Ocular Surface Disease Index (OSDI) [15]: 12 items assessing dry eye symptoms and vision-related quality of life. Total score ranges from 0 to 100, with severity categories as follows: 0–12 normal, 13–22 mild, 23–32 moderate, and 33–100 severe.

Internet Addiction Test (IAT) [16]: a 20-item scale (score range: 20–100); ≥ 50 indicates problematic internet use, and ≥80 indicates severe addiction.

Chinese Internet Addiction Scale—Revised (CIAS-R) [17]: 19 items assessing core symptoms of internet addiction (compulsion, withdrawal, tolerance, time management, and interpersonal/health problems). Score ranges from 19–76; higher scores indicate more severe symptoms.

Mobile Phone Addiction Index (MAPI) [18]: 24 items covering loss of control, withdrawal, avoidance, and productivity loss. Score ranges from 24–96; higher scores indicate greater addiction severity.

The CIAS-R total score ranges from 19 to 76, with higher scores indicating more severe internet addiction symptoms. The MAPI total score ranges from 24 to 96, similarly reflecting the degree of mobile phone addiction.

The CIAS-R and MAPI were originally validated in adolescent populations. However, both scales have been subsequently used in adult samples in several Chinese studies, and their internal consistency in our adult sample was acceptable (Cronbach’s α = 0.89 for CIAS-R and 0.91 for MAPI). Nonetheless, we acknowledge that the psychometric properties may differ across age groups, and this should be considered when interpreting the results.

### 2.3. Statistical Analysis

Data were analysed using IBM SPSS 27.0 and R 4.3.1 (ggplot2, corrplot). Normality was assessed using Shapiro–Wilk test; non-normal variables were log-transformed or analysed with non-parametric tests (Kruskal–Wallis). Group comparisons were performed using one-way ANOVA with Tukey’s post hoc test, or Kruskal–Wallis tests with Dunn’s test. For correlation analyses, Spearman’s rank correlation coefficients were used for non-normally distributed variables to account for the ordinal nature of several scales. For multiple linear regression models, we assessed residual normality (Shapiro–Wilk test), homoscedasticity (Breusch–Pagan test), and influential cases (Cook’s distance > 1). All models met these assumptions. Dose–response relationships were examined using linear regression and segmented linear regression to identify thresholds. A two-sided *p* < 0.05 was considered statistically significant. In multiple linear regression models, variance inflation factors (VIF) were calculated to assess multicollinearity. All VIF values remained below 5, indicating acceptable collinearity among predictors. For pairwise comparisons (Table 3), effect sizes were calculated as Cohen’s d using pooled standard deviations. Effect sizes were interpreted as small (d ≥ 0.2), medium (d ≥ 0.5), or large (d ≥ 0.8). For multiple comparisons across multiple outcome measures, we applied the Benjamini–Hochberg false discovery rate (FDR) correction to control for type I error. All significant results reported remained significant after FDR correction (q < 0.05).

To further address the potential confounding introduced by the asymmetric grouping strategy (i.e., the non-addicted group required both short-video duration < 0.5 h/day and IAT < 50, whereas the short-video group was defined solely by duration ≥ 1 h/day), we performed a post hoc sensitivity analysis. Within the short-video group (*n* = 164), participants were stratified according to their IAT scores into a high-addiction subgroup (IAT ≥ 50, indicating problematic internet use) and a low-addiction subgroup (IAT < 50). We compared the health outcomes across three groups: (1) the non-addicted control group (*n* = 164), (2) the low-addiction subgroup, comprising heavy viewers without significant internet addiction, and (3) the high-addiction subgroup, comprising heavy viewers with comorbid internet addiction. Due to the limited sample size in the low-addiction subgroup, we primarily report descriptive comparisons (means and mean differences) without inferential testing to avoid overinterpretation of statistically unstable estimates. This sensitivity analysis aimed to disentangle the independent effect of prolonged viewing duration from the concurrent effect of internet addiction severity.

## 3. Results

### 3.1. Participant Characteristics

The final sample comprised 328 young adults (mean age 24.9 ± 2.9 years; range 20–33 years; 55.2% female). No significant differences between the non-addicted group (*n* = 164) and the short-video group (*n* = 164) were found for age, gender, or region (all *p* > 0.05). The short-video group had a significantly shorter mean sleep duration than the non-addicted group (6.8 ± 1.1 h vs. 8.3 ± 0.9 h, t = 13.52, *p* < 0.0001).

The descriptive statistics of all participants stratified by daily short-video duration are summarised in Table 1. Sleep duration decreased progressively from 8.31 ± 0.88 h in the non-addicted group to 5.65 ± 1.35 h in the > 6 h group. Similarly, HADS, VDAS, and OSDI scores increased monotonically with longer daily short-video viewing duration (all *p* < 0.0001, one-way ANOVA).

Table 2 summarizes all clinical characteristics. The short-video group consistently showed higher scores on HADS, VDAS, OSDI, IAT, CIAS-R, and MAPI, as well as lower SF-36 PCS and MCS scores (all *p* < 0.0001).

### 3.2. Dose–Response Relationships Between Daily Short-Video Duration and Health Outcomes

**Figure 1:**
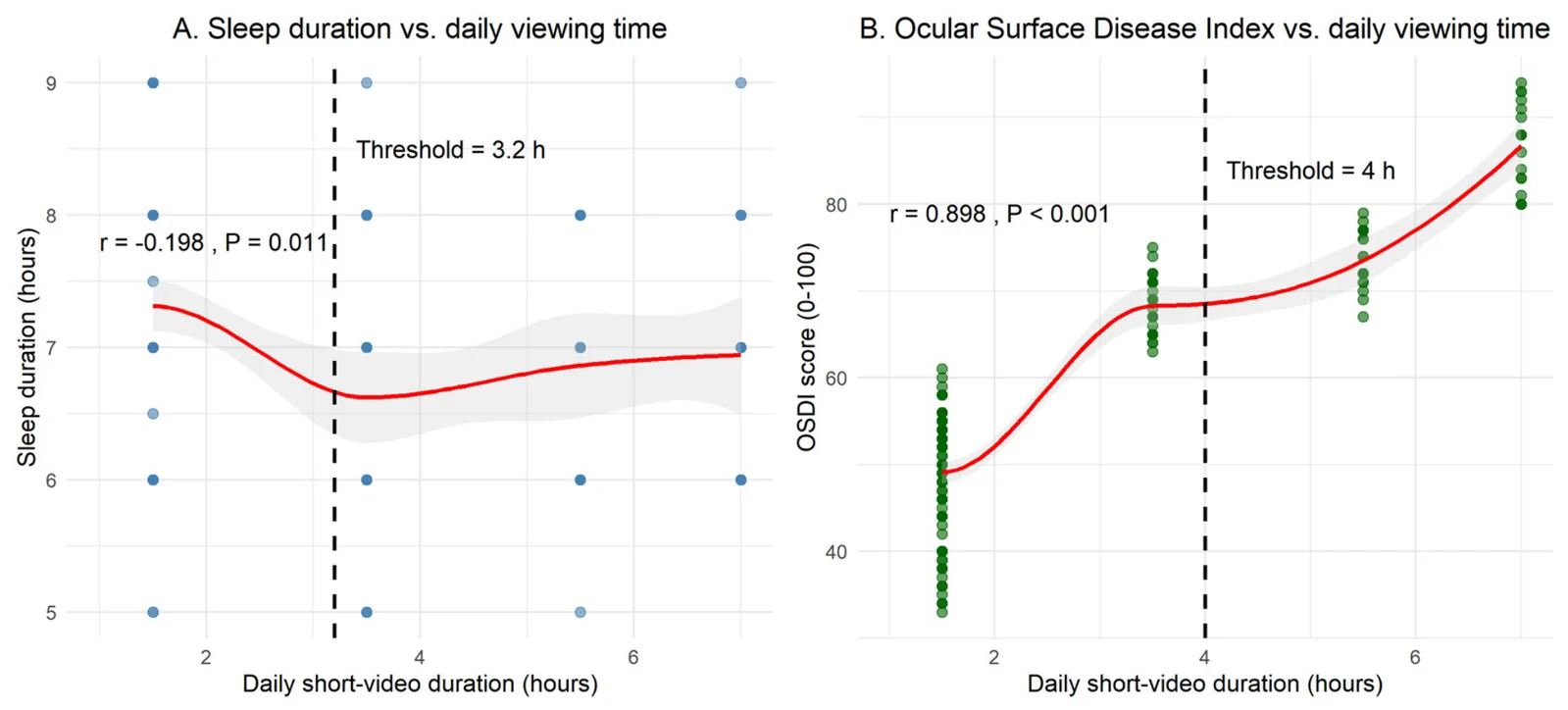
Dose–response relationships with segmented regression thresholds (black dashed lines). In each panel, individual data points are colour-coded by viewing duration (blue: 1–2 h; green: 3–4 h; orange: 5–6 h; red: >6 h). The solid red line represents the LOWESS smoothed trend, and the red dashed lines indicate the 95% confidence interval of the smoothed estimate. The black dashed vertical line marks the exploratory threshold identified by segmented regression. (**A**) Sleep duration vs. daily short-video duration (threshold = 3.2 h/day). (**B**) OSDI score vs. daily short-video duration (threshold = 4.0 h/day). Red lines: LOWESS smooth with 95% CI. Pearson r and *p* values are shown in each panel. Each point represents one participant in the longer-viewing group (*n* = 164). Shaded areas represent 95% CI; thresholds are exploratory due to limited sample size in high-duration groups (3–4 h: *n* = 29; 5–6 h: *n* = 22; >6 h: *n* = 17). Therefore, the suggested breakpoints should be interpreted with caution.

Figure 1 illustrates the dose–response relationships. Sleep duration decreased linearly with increasing daily short-video viewing duration (r = –0.73, 95% CI: –0.79 to –0.66, *p* < 0.001). Segmented regression identified a potential threshold at approximately 3.2 h/day: below this value, sleep duration declined modestly (slope = –0.21 h per additional hour); above 3.2 h, the slope steepened to –0.58 h per hour (*p* for slope difference <0.01).

The OSDI score increased monotonically with daily viewing duration (r = 0.89, *p* < 0.001). A threshold was suggested at around 4.0 h/day, with a steeper rise beyond that point. Among participants viewing > 6 h/day, the mean OSDI reached 86.5 ± 4.8, corresponding to severe dry eye symptoms. It should be noted that the threshold estimates are based on relatively small sample sizes in the higher duration groups (3–4 h: *n* = 29; 5–6 h: *n* = 22; >6 h: *n* = 17); therefore, these breakpoints should be interpreted with caution.

Table 3 reports pairwise comparisons between adjacent duration groups. The largest mean differences were observed for OSDI between the 1–2 h and 3–4 h groups (Δ = 20.2, 95% CI: 14.8–25.6, *p* < 0.001), and for HADS between the 1–2 h and 3–4 h groups (Δ = 1.9, 95% CI: 1.4–2.4). Notably, all comparisons for OSDI and HADS yielded large effect sizes (Cohen’s d ≥ 0.58, most > 1.7).

**Figure 2:**
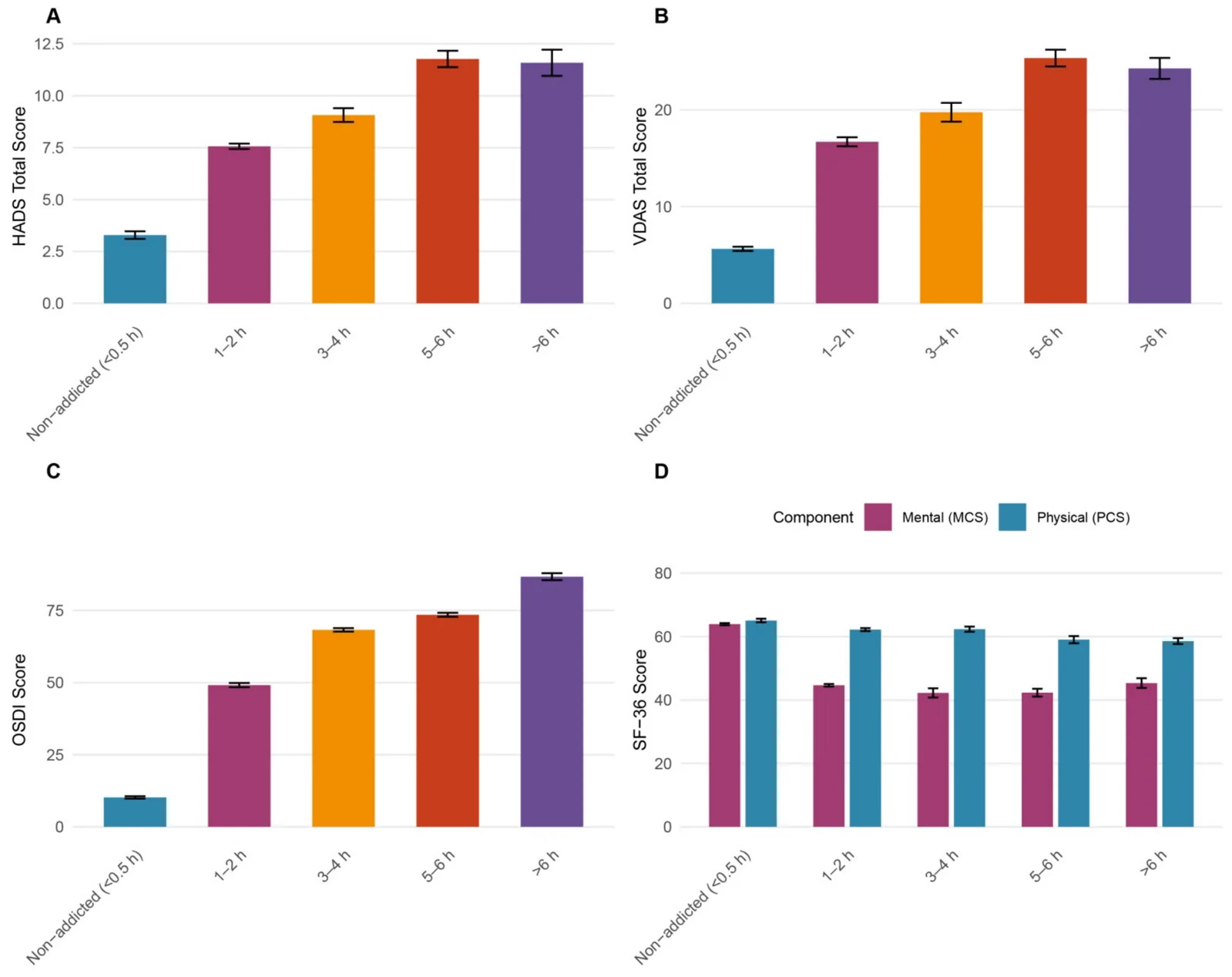
Comparison of health indicators across short-video duration groups. Grouped bar charts showing mean ± SEM for four outcome measures across five duration groups (Non-addicted < 0.5 h, *n* = 164; 1–2 h, *n* = 96; 3–4 h, *n* = 29; 5–6 h, *n* = 22; >6 h, *n* = 17). (**A**) Hospital Anxiety and Depression Scale (HADS, total score). (**B**) Van Dream Anxiety Scale (VDAS, total score). (**C**) Ocular Surface Disease Index (OSDI). (**D**) SF-36 Physical Component Summary (PCS) and Mental Component Summary (MCS), shown as separate side-by-side bars with standard error bars. PCS and MCS are reported as distinct constructs and are not summed into a total score. One-way ANOVA: all *p* < 0.0001. Tukey’s post hoc comparisons confirmed that each short-video group (1–2 h to >6 h) had significantly worse scores than the non-addicted group (*p* < 0.001 for all). Error bars = SEM.

Figure 2 compares HADS, VDAS, OSDI, and SF-36 PCS and MCS scores across the five duration groups. All four measures showed significant between-group differences (one-way ANOVA, *p* < 0.0001). Post hoc comparisons indicated that the 3–4 h group already had significantly higher HADS and OSDI scores than the 1–2 h group (*p* < 0.01), and the > 6 h group exhibited the poorest outcomes (all pairwise comparisons vs. non-addicted, *p* < 0.0001).

### 3.3. Correlation Matrix Highlighting Relationships with Mobile Phone Addiction

**Figure 3:**
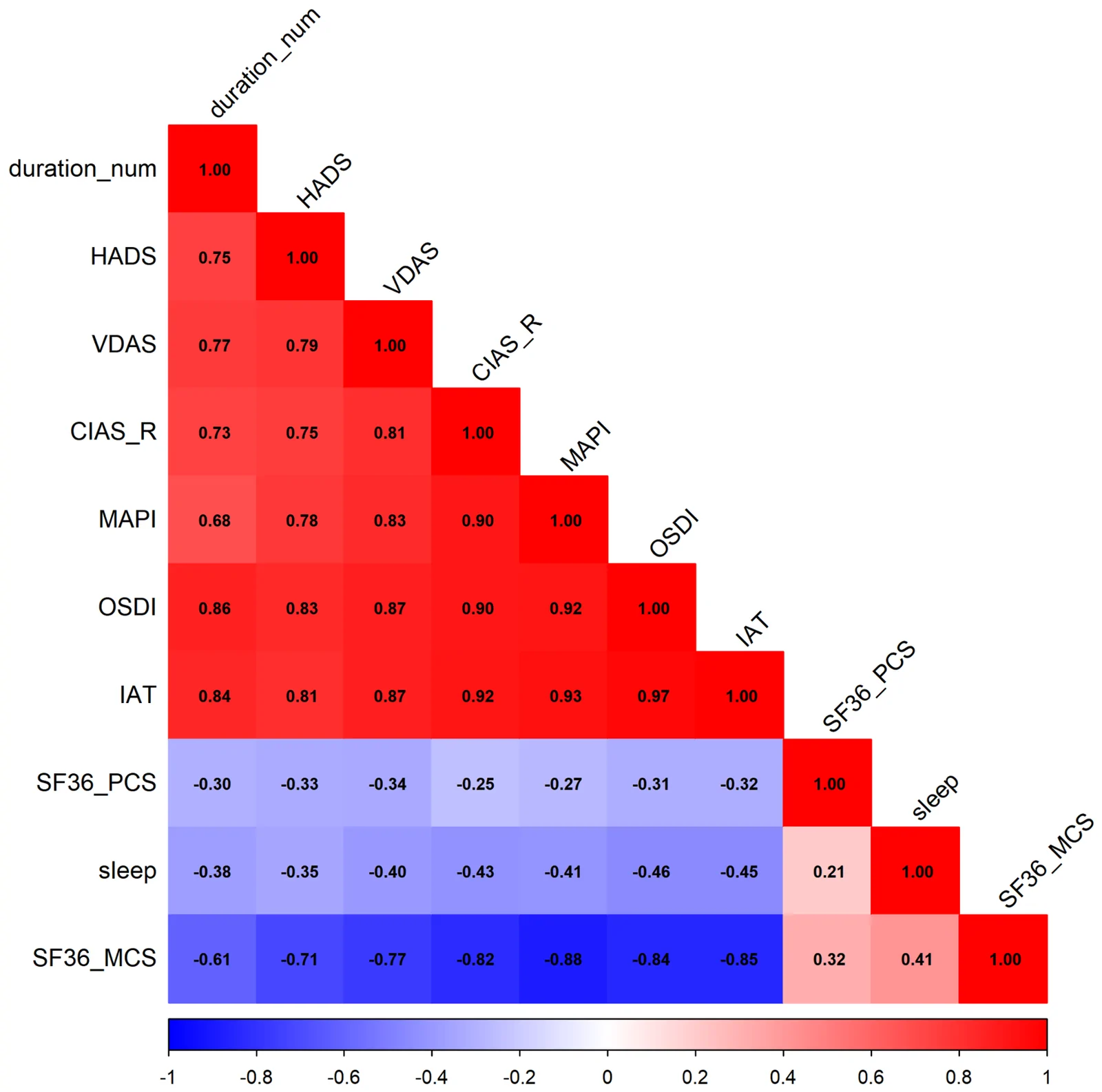
Correlation heatmap of all study variables. Spearman correlation matrix for continuous variables (daily short-video viewing duration, sleep duration, HADS, VDAS, OSDI, SF-36 PCS, SF-36 MCS, IAT, CIAS-R, MAPI). The colour scale ranges from blue (positive correlation) to red (negative correlation). Correlation coefficients are displayed in each cell when |r| > 0.3. The strongest correlations are highlighted: MAPI with VDAS (r = 0.77), duration with OSDI (r = 0.89), and duration with sleep (r = −0.73). Gender and age were not included in the heatmap as they showed no significant correlations with the main outcomes (all *p* > 0.05). The correlation between CIAS-R and MAPI is also strong (r = 0.71), indicating overlapping but not identical constructs. All displayed correlations (|r| > 0.3) were significant at *p* < 0.001.

As shown in the correlation heatmap (Figure 3), MAPI was strongly correlated with VDAS (r = 0.77, *p* < 0.001), which was higher than the correlations between IAT and VDAS (r = 0.62) and between CIAS-R and VDAS (r = 0.68). OSDI correlated most strongly with daily viewing duration (r = 0.89), followed by MAPI (r = 0.74) and CIAS-R (r = 0.71). Sleep duration showed strong negative correlations with MAPI (r = –0.70) and IAT (r = –0.65). SF-36 PCS was inversely associated with CIAS-R (r = –0.58) and MAPI (r = –0.61).

Notably, no significant gender differences were observed for any of the health outcomes or addiction scales (all *p* > 0.05). The strong correlation between MAPI and VDAS (r = 0.77) is consistent with the hypothesis that mobile phone addiction, particularly pre-sleep use, may be associated with dream anxiety. However, consistent with our cross-sectional design, we cannot infer causality from this correlation.

### 3.4. Prevalence of Clinically Significant Conditions by Duration Group

**Figure 4:**
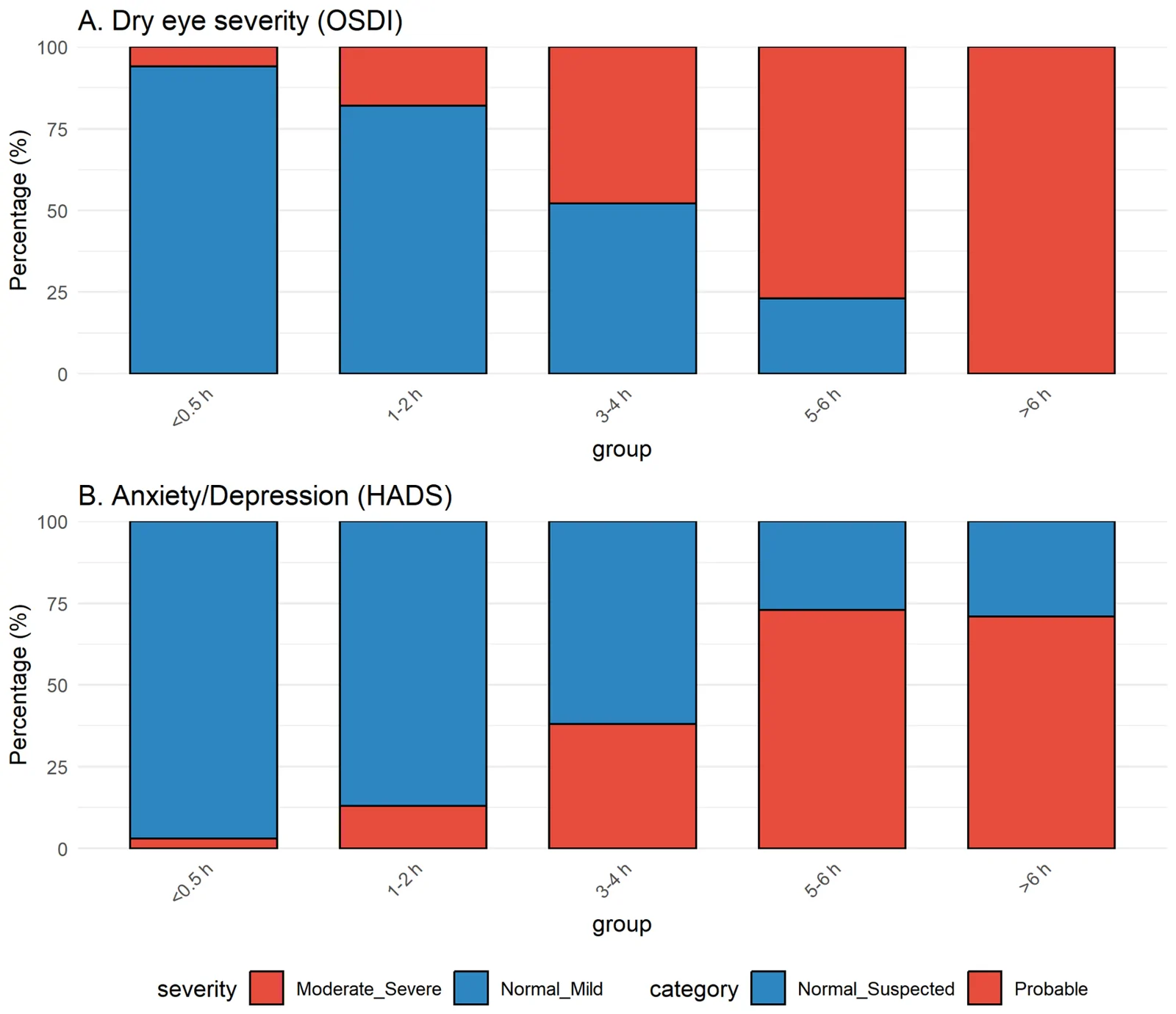
Prevalence of clinically significant conditions by short-video duration group. Stacked bar charts showing the proportion of participants in each risk category across duration groups. (**A**) Dry eye severity based on OSDI: Normal (0–12), Mild (13–22), Moderate-to-Severe (≥23). (**B**) Anxiety/depression based on HADS: Normal (0–7), Suspected (8–10), Probable (≥11). The proportion of participants with moderate-to-severe dry eye increased from 6% in the non-addicted group to 100% in the >6 h group. Similarly, the proportion with probable anxiety/depression rose from 3% to 71%. The steepest increases occurred between the 1–2 h and 3–4 h groups for HADS, and between the 3–4 h and 5–6 h groups for OSDI. The exact sample sizes per group: non-addicted *n* = 164, 1–2 h *n* = 96, 3–4 h *n* = 29, 5–6 h *n* = 22, >6 h *n* = 17. Percentages are based on these group sizes.

Figure 4 displays stacked bar charts showing the proportion of participants with moderate-to-severe dry eye (OSDI ≥ 23) and probable anxiety/depression (HADS ≥ 11). Among the non-addicted group, only 6% had moderate-to-severe dry eye and 3% had HADS ≥ 11. In the 1–2 h group, these proportions rose to 18% and 12%, respectively. In the 3–4 h group, they reached 47% and 38%, respectively. In the 5–6 h group, the corresponding proportions were 76% and 71%, respectively. Among participants viewing > 6 h/day, all (100%, 95% CI: 80.5–100%) had moderate-to-severe dry eye (OSDI ≥ 23), and 71% (12/17, 95% CI: 44.0–89.7%) had probable anxiety/depression (HADS ≥ 11). Even in the non-addicted group, a small proportion of participants (6%) had moderate-to-severe dry eye, possibly attributable to screen time from other devices.

### 3.5. Regression Analyses

Multiple linear regression analyses (Table 4) showed that, after adjusting for age, gender, and region, daily short-video duration (standardised β = 0.62, *p* < 0.001) and MAPI score (β = 0.35, *p* < 0.01) were associated with OSDI (R^2^ = 0.74). For HADS, the factors significantly associated with the outcome included daily short-video duration (β = 0.48, *p* < 0.001) and CIAS-R score (β = 0.29, *p* < 0.05; R^2^ = 0.68). For SF-36 PCS, duration (β = –0.44, *p* < 0.001) and sleep duration (β = 0.38, *p* < 0.001) together explained 55% of the variance. Despite the relatively high R^2^ values, variance inflation factors for all predictors remained below 5, indicating acceptable collinearity. The strong association between duration and MAPI (r = 0.74) warrants caution, but the VIF values suggest that multicollinearity did not substantially distort the regression estimates.

### 3.6. Sensitivity Analysis: Stratifying the Short-Video Group by IAT

Within the short-video group (*n* = 164), only 7 participants (4.3%) had IAT scores below 50 (low-addiction subgroup), while 157 participants (95.7%) had IAT scores of 50 or higher (high-addiction subgroup). The low-addiction subgroup comprised individuals who engaged in heavy daily short-video viewing (≥1 h/day) but did not meet the IAT threshold for problematic internet use, allowing us to observe the pattern of associations potentially related to viewing duration itself, albeit with very limited statistical power.

Descriptive comparisons between the low-addiction subgroup and the non-addicted control group revealed that heavy viewers without IAT-defined addiction still exhibited substantially worse outcomes across most metrics: mean OSDI was 35.3 (vs. 16.3 in controls), mean VDAS was 15.4 (vs. 5.2), and mean HADS was 8.0 (vs. 4.1), while mean sleep duration was comparable (8.14 h vs. 8.31 h). These descriptive patterns suggest that prolonged viewing duration itself, independent of meeting the IAT addiction threshold, may be associated with poorer ocular surface, dream anxiety, and psychological health indicators, although the small sample size (*n* = 7) precludes definitive statistical inference.

When comparing the high-addiction subgroup against the low-addiction subgroup, the high-addiction subgroup descriptively showed shorter sleep (mean difference ≈–1.4 h), higher OSDI (≈+19.5), higher VDAS (≈+8.8), and higher HADS (≈+9.3). This pattern indicates that concurrent internet addiction (IAT ≥ 50) may further exacerbate the adverse associations beyond the effect of duration alone.

Collectively, this sensitivity analysis, though exploratory and constrained by the small number of pure heavy viewers without IAT-diagnosed addiction, suggests that the duration of short-video viewing remains a relevant correlate of health outcomes even among participants without problematic internet use, while co-occurring internet addiction appears to amplify these associations. These findings should be interpreted with caution and await validation in larger samples with balanced subgroup sizes. The primary value of this analysis is to highlight the need for future research designed specifically to disentangle the independent effects of viewing duration from those of comorbid internet addiction.

## 4. Discussion

This study adds to the growing body of evidence linking short-video viewing in young adults to dose-dependent relationships with sleep, ocular surface health, dream-related anxiety, and quality of life. Several findings merit emphasis.

### 4.1. Ocular Surface Associations: Comparisons with Gaming Addiction Findings

The correlation between daily viewing duration and OSDI (r = 0.89) is substantially strong. In our previous gaming study, the correlation between game duration and OSDI was r = 0.68. Unlike gaming, which involves dynamic visual tracking and active task engagement, short-video viewing requires sustained central fixation with minimal saccades—a predominantly passive viewing mode that may exacerbate accommodative lag and tear film instability. The difference may be explained by: (i) blink rate suppression during short-video watching, which is even lower than during gaming due to rapid scene cuts [19]; (ii) shorter viewing distance (handheld phones vs. larger screens); (iii) environmental factors—watch short-videos in dim or lying-down positions, worsening tear film distribution. Preclinical studies have shown that chronic disruption of tear film homeostasis can be modelled pharmacologically, e.g., via topical erlotinib [20], supporting the biological plausibility of dry eye development from sustained blink suppression. Moreover, >6 h/day viewers had a mean OSDI score of 86.5 (±4.8), which exceeds the threshold for severe dry eye and is notably higher than previously reported in gaming addiction studies [21]. Such severe ocular surface disease may involve central pain processing, as patients with acute eye pain exhibit altered spontaneous brain activity in pain-related regions [22]. Clinicians may therefore consider screening for dry eye in young adults presenting with high short-video usage. Nevertheless, the cross-sectional design precludes causal attribution, and the strong correlation may partly reflect reverse causality (e.g., individuals with pre-existing dry eye might prefer passive screen activities). Additionally, the strength of this correlation may be partially inflated by shared method variance, as both exposure (viewing duration) and outcome (OSDI) were assessed via self-report questionnaires. Furthermore, given the asymmetric grouping strategy discussed in Methods 2.1, this association should be interpreted as reflecting a composite pattern of prolonged short-video viewing accompanied by heightened internet/mobile phone dependency, rather than an isolated effect of viewing time per se.

### 4.2. Dream Anxiety and the Mobile Phone Addiction Link

A novel finding is the strong correlation between MAPI and VDAS (r = 0.77). The Mobile Phone Addiction Index captures behaviours such as “feeling anxious when unable to check the phone” and “using phone before sleep.” Pre-sleep short-video viewing exposes viewers to blue light and emotionally arousing content (e.g., suspense, horror, or intensely funny videos), which may delay REM sleep onset and has been hypothesised to increase nightmare frequency [23]. Furthermore, the infinite-scroll design may induce a state of “micro-dissociation,” making it harder to disengage from the device, thereby encroaching on sleep time and fragmenting sleep architecture [24]. This aligns with recent neuroimaging findings that short-video viewing has been associated with alterations in frontostriatal circuits involved in reward and sleep regulation [25]. Moreover, the Chinese Internet Addiction Scale (CIAS-R) was also associated with HADS scores, supporting the notion that the broader construct of internet addiction, as captured by CIAS-R, extends beyond mobile-specific behaviours to affect mental health. Beyond blue light exposure, the psychological mechanisms of algorithm-driven infinite scrolling may play a critical role. The continuous delivery of novel, emotionally charged content triggers intermittent reward-seeking behaviour, which maintains high cortical arousal and may suppress the natural nocturnal rise of melatonin. This sustained cognitive activation not only delays sleep onset but also fragments rapid eye movement (REM) sleep architecture, a phase critically involved in emotional memory consolidation. Fragmented REM sleep has been linked to increased nightmare frequency in prior research, offering a plausible but untested explanation for our findings and dream-related anxiety [10]. Critically, the observed MAPI-VDAS correlation, like all associations in this study, derives from a comparison between groups that differ simultaneously in short-video duration and broader digital addiction severity. Thus, this finding should be viewed as an association with a composite exposure pattern rather than a specific effect of mobile phone addiction independent of short-video use. Thus, the association between MAPI and VDAS likely reflects a dual pathway—circadian disruption mediated by blue light exposure and cognitive hyperarousal driven by algorithm-guided content delivery. However, these proposed mechanisms remain speculative and require direct neurophysiological testing in future studies. All mechanistic interpretations presented above are speculative and are offered as hypothesis-generating explanations rather than as established pathways. Direct measurement of these variables would be required to test these mechanisms empirically.

### 4.3. Exploratory Dose–Response Patterns and Potential Clinical Considerations

We identified inflection points at approximately 3 h/day for sleep deterioration and 4 h/day for dry eye exacerbation. However, these thresholds are derived from a limited number of participants in the higher duration categories (e.g., only 17 participants viewed > 6 h/day, and the 3–4 h and 5–6 h groups comprised 29 and 22 participants, respectively). Therefore, the identified breakpoints should be considered exploratory and interpreted with caution until replicated in larger samples. The proportion of young adults with probable anxiety/depression (HADS ≥ 11) was substantially higher among those viewing >3 h/day (38%) compared to those viewing < 1 h/day (3%). Although our segmented regression identified exploratory thresholds at approximately 3.2 h/day for sleep and 4.0 h/day for OSDI, these values did not directly support a specific prescriptive target such as ≤1 h. The choice of any specific cut-off would require independent empirical justification and should not be inferred from our exploratory breakpoints. Our results are consistent with the “Goldilocks” hypothesis for screen time [11], which suggests moderate use (≤1 h) is not harmful, but excessive use (>3 h) is detrimental. The pairwise effect sizes for OSDI and HADS were large (Cohen’s d ranging from 0.58 to 1.88), further supporting that the observed differences between adjacent duration groups are not only statistically significant but also clinically relevant. These thresholds should not be used as clinical cut-offs until validated in large, prospective cohorts with adequate power for subgroup analyses. It is important to note that these exploratory thresholds should not be conflated with clinical or public health recommendations. The ≤1-h target, which has been suggested by some previous studies on general screen time, represents a distinct and more conservative hypothesis. Our data do not directly support or refute this specific cut-off; rather, they indicate that significant deteriorations in sleep and ocular surface health were observed at levels around 3–4 h. Future interventional trials are urgently needed to compare the efficacy and feasibility of different reduction targets (e.g., ≤1 h, ≤2 h, ≤3 h) in improving health outcomes.

### 4.4. Comparison with Gaming Addiction: Similarities and Differences

The patterns observed in this study share similarities with those reported for gaming addiction, but also exhibit notable differences. Our findings should be interpreted in the context of the ongoing active-versus-passive screen-use debate, as short-video consumption is predominantly passive and algorithm-guided, which may be associated with distinct risk patterns compared to active screen activities. However, it must be reiterated that the observed differences between the non-addicted and short-video groups may be attributable to a composite of longer duration and greater internet/mobile phone addiction severity, not to short-video duration alone. Two notable differences emerged: (i) Gender—our gaming study found males were significantly more addicted and had worse outcomes, whereas short-video viewing showed no gender difference, reflecting the universal appeal of short-videos across genders. (ii) Ocular surface—short-video viewing produced a stronger OSDI correlation (0.89 vs. 0.68). This may be due to the static near-work nature of short-videos: while gaming involves dynamic visual tracking and occasional distance viewing (e.g., cutscenes), short-videos keep the eyes fixed in a small central area with minimal saccades, increasing accommodative lag and dry eye risk [26]. The absence of significant gender differences in this study contrasts with our previous findings on gaming addiction, where males showed higher addiction severity and worse health outcomes. This null finding may reflect the algorithm-driven content of short-video platforms, which appeals broadly to both genders, unlike the more gender-stereotyped gaming genres. However, given the limited sample size, we cannot rule out a type II error; this observation warrants replication in larger, more diverse samples.

### 4.5. Limitations

Several limitations should be acknowledged, which warrant careful consideration when interpreting the findings.

First, a major overarching limitation is the potential for substantial residual confounding. Our regression models adjusted only for age, gender, and region. We did not measure or account for several critical confounders, including total screen time across all devices (e.g., smartphones, computers, tablets), concurrent gaming behaviour, physical activity, socioeconomic status (e.g., education, occupation, income), body mass index, caffeine or alcohol intake, sleep disorders, contact lens use, and pre-existing dry eye or other ocular conditions. Consequently, the observed associations, while statistically strong, may be partially or largely explained by these unmeasured factors. For instance, an individual who spends 4 h daily on short-video platforms, 4 h on a computer, and additional time on other devices may have elevated OSDI scores primarily due to cumulative screen exposure rather than short-video viewing specifically. Likewise, we cannot rule out the possibility that concurrent gaming behaviour confounds the observed relationships, as gaming time was not included as a covariate in our regression models. Furthermore, reverse causality is a plausible alternative explanation: individuals with pre-existing dry eye, anxiety, or sleep disturbances may preferentially engage in passive, low-effort screen activities such as short-video viewing, rather than the viewing causing these conditions. Therefore, our findings should be considered hypothesis-generating rather than confirmatory, and await rigorous testing in future studies with comprehensive covariate assessment and longitudinal designs.

Second, the cross-sectional design precludes any causal inference. The temporal sequence between short-video viewing and the measured health outcomes cannot be established. While we have discussed biologically plausible mechanisms, including blue-light exposure, reduced blink frequency, cognitive hyperarousal, and REM sleep disruption, these interpretations remain speculative. Our study did not directly measure any of these physiological or neurocognitive variables. Thus, all mechanistic interpretations are offered as hypothesis-generating explanations rather than as established pathways, and require direct neurophysiological testing in future experimental or interventional studies.

Third, the asymmetric grouping strategy introduces interpretative difficulties that cannot be fully resolved. The non-addicted group was defined using both low viewing duration (<0.5 h/day) and a low IAT score (<50), whereas the short-video group was defined solely by viewing duration (≥1 h/day) without an IAT restriction. As shown in Table 2, the two groups differ simultaneously in both short-video duration and broader internet/mobile phone addiction severity (IAT, CIAS-R, and MAPI scores). Consequently, the observed differences should be interpreted as reflecting a composite exposure pattern—prolonged short-video viewing accompanied by elevated smartphone/internet dependency—rather than an isolated effect of viewing duration per se. Although we conducted a post hoc sensitivity analysis stratifying the short-video group by IAT score to explore this issue, this analysis was severely underpowered (only 7 participants fell into the low-addiction subgroup) and could not definitively disentangle the independent contributions of duration versus addiction. This remains a fundamental limitation of the current study design, and future investigations should recruit adequately sized samples with balanced subgroup distributions to address this question directly.

Fourth, all primary exposures and outcomes were assessed using self-report questionnaires. This reliance introduces several forms of bias, including recall bias, social desirability bias, and—most importantly—common-method bias. The strong correlations observed (e.g., daily viewing duration vs. OSDI, r = 0.89) may be partially inflated by shared method variance, as both the exposure and outcome variables were derived from the same self-report instruments. We lacked objective measures of short-video viewing time (e.g., app usage logs), blink rate, tear film breakup time (objective dry eye assessment), actigraphy or polysomnography (objective sleep assessment), or clinical psychiatric evaluation. Future studies should incorporate such objective assessments to strengthen physiological validity and reduce bias.

Fifth, the dose–response threshold analyses should be interpreted with utmost caution. The segmented regression identified exploratory breakpoints at approximately 3.2 h/day for sleep deterioration and 4.0 h/day for OSDI exacerbation. However, these estimates are derived from limited sample sizes in the higher duration categories (3–4 h: *n* = 29; 5–6 h: *n* = 22; >6 h: *n* = 17), resulting in wide confidence intervals and unstable model fits. These thresholds should not be interpreted as clinically meaningful cut-points or evidence-based behavioural prescriptions. They are exploratory observations that require validation in large, prospective cohorts with adequate statistical power for subgroup analyses. Moreover, these thresholds do not directly support or refute any specific duration target (e.g., ≤1 h/day), and should not be conflated with public health recommendations.

Sixth, the generalizability of our findings is limited. The sample was confined to Chinese young adults aged 20–33 years, recruited via convenience sampling through social media platforms. This age- and culture-restricted sample may not represent other age groups, adolescents, older adults, or populations from different cultural or socioeconomic backgrounds. Additionally, the convenience sampling method may introduce selection bias, although we attempted to minimize this by not disclosing specific hypotheses in the recruitment advertisement.

Seventh, the psychometric properties of the CIAS-R and MAPI warrant consideration. Both scales were originally validated in adolescent populations. Although their internal consistency was acceptable in our adult sample (Cronbach’s α= 0.89 and 0.91, respectively), the factor structure and measurement invariance may differ across age groups. Future studies should validate these instruments specifically in adult populations or utilize scales with established adult norms.

Eighth, several additional methodological limitations should be noted. We did not conduct a formal a priori power analysis; post hoc power estimates should be interpreted cautiously, and subgroup analyses were underpowered. The Benjamini-Hochberg FDR correction was applied to control for multiple comparisons, and all significant results remained significant after correction (q < 0.05); nonetheless, the large number of outcomes and comparisons performed increases the risk of type I error. Finally, although we excluded participants with incomplete or inconsistent responses, and comparisons between included and excluded participants showed no significant differences, we cannot fully rule out non-response bias.

In summary, the findings of this study are best viewed as exploratory and hypothesis-generating. They provide preliminary evidence for dose–response-like associations between short-video viewing and multiple health domains in young adults, but causal conclusions, clinical recommendations, and specific duration targets cannot be drawn from the current data. Future longitudinal and interventional studies with comprehensive covariate adjustment, objective exposure and outcome measurements, and adequately powered subgroup analyses are urgently needed to confirm these associations and establish temporal sequences.

## 5. Conclusions

Prolonged daily short-video viewing among young adults is associated with progressively shorter sleep duration in a dose-dependent manner, poorer ocular surface health, greater dream-related anxiety, and lower overall quality of life. The relationships with dry eye and dream anxiety are particularly apparent, likely due to the specific characteristics of short-video engagement: close-range handheld viewing, reduced blinking, and pre-sleep usage. Importantly, the strong association between mobile phone addiction (MAPI) and dream anxiety underscores the need to address bedtime phone use.

Primary care physicians and ophthalmologists should consider screening for excessive short-video use in young adults presenting with unexplained dry eye, sleep disturbances, or anxiety symptoms. When managing individuals who exhibit severe dependency, clinical strategies should consider structured mental health frameworks, drawing on established comparative research indicating that cognitive-behavioural therapies often offer superior long-term behavioural modification compared to standard psychoeducational limit-setting alone [27].

Future interventional studies are needed to identify the optimal duration limit. Our exploratory thresholds (approximately 3.2 h/day for sleep and 4.0 h/day for OSDI) provide preliminary reference points, but the specific target duration remains to be determined through adequately powered studies designed to compare multiple cut-off levels. These reference points should not be interpreted as evidence-based behavioural prescriptions without validation in prospective, interventional settings.

## Figures and Tables

**Table 1:** Descriptive characteristics of participants stratified by daily short-video duration.

Group (Daily Viewing Duration)	*n*	Sleep Duration (h)	HADS Total	VDAS Total	OSDI Total	SF-36 PCS	SF-36 MCS
Non-addicted (<0.5 h)	164	8.31 ± 0.88	4.12 ± 2.45	5.23 ± 2.12	16.34 ± 5.21	67.3 ± 6.10	64.5 ± 5.20
1–2 h	96	7.20 ± 0.91	14.23 ± 3.81	19.51 ± 6.22	48.27 ± 11.18	53.2 ± 6.50	41.3 ± 7.10
3–4 h	29	6.50 ± 1.06	21.72 ± 4.53	27.31 ± 7.09	68.48 ± 9.78	43.6 ± 5.80	31.5 ± 6.40
5–6 h	22	6.00 ± 1.28	26.41 ± 5.18	34.14 ± 8.27	73.91 ± 8.70	38.7 ± 4.90	25.9 ± 5.10
>6 h	17	5.65 ± 1.35	28.86 ± 5.77	41.18 ± 9.63	86.53 ± 4.82	34.2 ± 4.60	21.7 ± 4.80

Note: Data are presented as mean ± SD. HADS = Hospital Anxiety and Depression Scale (total score); VDAS = Van Dream Anxiety Scale; OSDI = Ocular Surface Disease Index; SF-36 PCS = Physical Component Summary; SF-36 MCS = Mental Component Summary. PCS and MCS are reported separately, as they represent distinct constructs and are not recommended to be summed into a single total score. One-way ANOVA revealed significant differences across all five groups for every outcome (all *p* < 0.0001). Post hoc comparisons versus the non-addicted group were significant at *p* < 0.001 for all short-video groups.

**Table 2:** Demographic and clinical characteristics of participants (mean ± SD or *n*%).

Variable	Non-Addicted (*n* = 164)	Short-Video (*n* = 164)	*p* Value
Age (years)	24.7 ± 2.9	25.1 ± 2.8	0.21
Gender (male, %)	45.5%	44.2%	0.82
Region (northern, %)	58.2%	60.6%	0.66
Sleep duration (h)	8.31 ± 0.88	6.79 ± 1.12	<0.0001
HADS total	4.12 ± 2.45	17.34 ± 5.67	<0.0001
VDAS total	5.23 ± 2.12	24.18 ± 7.23	<0.0001
OSDI total	16.34 ± 5.21	54.76 ± 14.89	<0.0001
SF-36 PCS	67.3 ± 6.1	53.2 ± 6.5	<0.0001
SF-36 MCS	64.5 ± 5.2	41.3 ± 7.1	<0.0001
IAT total	19.8 ± 4.1	59.4 ± 13.2	<0.0001
CIAS-R total	27.4 ± 5.3	64.5 ± 12.1	<0.0001
MAPI total	26.1 ± 4.9	72.3 ± 15.4	<0.0001

Note: Data are mean ± SD or *n* (%). Group comparisons: *t*-test or Mann–Whitney U test for continuous variables; χ^2^ test for categorical variables. HADS, Hospital Anxiety and Depression Scale (total score range 0–21 for each subscale, total 0–42); VDAS, Van Dream Anxiety Scale (range 0–48); OSDI, Ocular Surface Disease Index (range 0–100); SF-36 PCS and MCS, Physical and Mental Component Summaries of the 36-Item Short-Form Health Survey (each ranging from 0–100, higher = better); IAT, Internet Addiction Test (range 20–100); CIAS-R, Chinese Internet Addiction Scale—Revised (range 19–76); MAPI, Mobile Phone Addiction Index (range 24–96). All *p* values for group comparisons were <0.0001 except where indicated.

**Table 3:** Pairwise comparisons of health indicators across short-video duration groups, with Cohen’s d effect sizes.

Comparison	Δ OSDI (95% CI)	Cohen’s d	*p*	Δ HADS (95% CI)	Cohen’s d	*p*	Δ Sleep (h) (95% CI)	Cohen’s d	*p*
1–2 h vs. 3–4 h	+20.2 (14.8–25.6)	1.86	<0.001	+1.9 (1.4–2.4)	1.88	<0.001	−0.6 (−0.9–0.3)	–0.74	<0.01
3–4 h vs. 5–6 h	+5.4 (1.2–9.6)	0.58	<0.0001	+0.8 (0.3–1.3)	0.97	<0.01	−0.5 (−1.1–0.1)	–0.43	<0.001
5–6 h vs. >6 h	+12.6 (6.8–18.4)	1.73	<0.01	+1.5 (0.9–2.1)	0.45	<0.01	–0.4 (–0.7–0.1)	–0.27	<0.05

Note: Δ = mean difference (higher duration minus lower duration). Cohen’s d calculated using pooled standard deviation from the two compared groups. 95% CI in parentheses. Pairwise comparisons by *t*-test with Bonferroni correction (α = 0.0167). OSDI = Ocular Surface Disease Index; HADS = Hospital Anxiety and Depression Scale. Positive Cohen’s d for OSDI/HADS indicates worse outcome in the longer-duration group; negative d for sleep indicates shorter sleep in the longer-duration group.

**Table 4:** Multivariate linear regression models for OSDI, HADS, and SF-36 PCS.

Outcome	Predictor	β (Standardized)	95% CI	*p*	R^2^	Adjusted R^2^
OSDI	daily short-video viewing duration	0.62	0.51–0.73	<0.001	0.74	0.737
	MAPI	0.35	0.19–0.51	<0.01		
	Age	−0.05	−0.12–0.02	0.21		
HADS	daily short-video viewing duration	0.48	0.36–0.60	<0.001	0.68	0.677
	CIAS-R	0.29	0.12–0.46	<0.05		
	Sleep duration	−0.21	−0.38 to −0.04	<0.05		
SF-36 PCS	daily short-video viewing duration	−0.44	−0.57 to −0.31	<0.001	0.55	0.547
	Sleep duration	0.38	0.24–0.52	<0.001		

Note: β = standardized regression coefficient. 95% CI in brackets. Models adjusted for age, gender, and region. R^2^ = coefficient of determination. OSDI, Ocular Surface Disease Index; HADS, Hospital Anxiety and Depression Scale; SF-36 PCS, Physical Component Summary; MAPI, Mobile Phone Addiction Index; CIAS-R, Chinese Internet Addiction Scale—Revised.

## Data Availability

The datasets generated and analysed during the current study are available from the corresponding author on reasonable request. The data presented in this study are not publicly available due to privacy and ethical restrictions, as the study involved human participants and the informed consent obtained did not include provisions for public data sharing.
